# Comparison of patellofemoral osteoarthritis between patients treated surgically or non‐surgically following anterior cruciate ligament injuries: A systematic review and meta‐analysis

**DOI:** 10.1002/jeo2.70697

**Published:** 2026-05-18

**Authors:** Domenico Franco, Alexander Bumberger, Sebastian Schmidt, Chilan B. G. Leite, Fabrizio Russo, Gianluca Vadalà, Rocco Papalia, Vincenzo Denaro, Cale Jacobs, Christian Lattermann

**Affiliations:** ^1^ Department of Orthopedic Surgery Brigham and Women's Hospital, Harvard Medical School Boston Massachusetts USA; ^2^ Operative Research Unit of Orthopaedic and Trauma Surgery Fondazione Policlinico Universitario Campus Bio‐Medico Rome Italy; ^3^ Department of Orthopedics and Trauma Surgery Medical University of Vienna Vienna Austria; ^4^ Department of Orthopaedic and Trauma Surgery, University Medical Centre Mannheim, Medical Faculty Mannheim University of Heidelberg Mannheim Germany

**Keywords:** anterior cruciate ligament injury, anterior cruciate ligament reconstruction, bone patellar tendon bone autograft, hamstring autograft, patellofemoral osteoarthritis, post‐traumatic osteoarthritis

## Abstract

**Purpose:**

We hypothesized that patients undergoing anterior cruciate ligament reconstruction (ACL‐R) are more likely to develop patellofemoral osteoarthritis (PFOA) than those with an ACL injury managed non‐surgically.

**Study design:**

Systematic review and Meta‐analysis.

**Methods:**

A systematic literature review and meta‐analysis were performed in accordance with the Preferred Reporting Items for Systematic Reviews and Meta‐Analysis (PRISMA) guidelines. Studies reporting PFOA assessment by radiographic or magnetic resonance imaging after ACL‐R compared to non‐surgical treatment of ACL injury were included. Studies involving individuals with concurrent other ligamentous or chondral injuries treated surgically were excluded to minimize bias.

**Results:**

A total of six studies were included in the systematic review. The meta‐analysis demonstrated that patients undergoing ACL‐R had a significantly higher likelihood of developing PFOA compared to those who received non‐surgical treatment after ACL injury (odds ratio [OR] = 2.21; 95% confidence interval [CI]: 1.13−4.35; *p* = 0.02). Patients who underwent ACL‐R with bone–patellar tendon–bone (BPTB) autograft exhibited a significantly higher prevalence of PFOA than those with HT autograft (*p* < 0.01). Moreover, male patients exhibited a higher association with PFOA than females in both the ACL‐R (34.38% vs. 7.77%, *p* < 0.001) and non‐surgical ACL injury groups (28.13% vs. 0.97%, *p* < 0.001).

**Conclusions:**

ACL‐R significantly increases the likelihood of PFOA compared to non‐surgical ACL treatment, especially when using BPTB autografts. Male patients demonstrated a higher association with PFOA in both groups. Targeted preventive strategies, including optimized graft selection and enhanced rehabilitation protocols, are crucial for reducing the risk of PFOA following ACL‐R.

**Level of Evidence:**

Level III.

**Abbreviations**
ACL: anterior cruciate ligament
ACL‐R: anterior cruciate ligament reconstruction
BMI: body mass index
BPTB: bone–patellar tendon–bone (autograft)
CI: confidence interval
DOI: digital object identifier
HT: hamstring tendon (autograft)
ICOAP: Intermittent and Constant Osteoarthritis Pain Score
IKDC: International Knee Documentation Committee
IL‐1β: interleukin‐1 beta
IL‐6: interleukin‐6
KL: Kellgren and Lawrence (classification)
KOOS: Knee Injury and Osteoarthritis Outcome Score
LET: lateral extra‐articular tenodesis
MCMS: Modified Coleman Methodology Score
MRI: magnetic resonance imaging
*N*
: number
OA: osteoarthritis
OARSI: Osteoarthritis Research Society International
OR: odds ratio
PFJ: patellofemoral joint
PFOA: patellofemoral osteoarthritis
PRISMA: Preferred Reporting Items for Systematic Reviews and Meta‐Analysis
PRO: patient‐reported outcomes
PTOA: post‐traumatic osteoarthritis
RCT: randomized controlled trial
ROBINS‐I: Risk of Bias in Non‐randomized Studies of Interventions
RoB 2: Risk of Bias Tool Version 2
SF‐36: 36‐Item Short Form Survey
TNF‐α: tumor necrosis factor‐alpha

## INTRODUCTION

Anterior cruciate ligament (ACL) injury is a common musculoskeletal condition, particularly among young and physically active individuals, often resulting from pivoting or high‐impact sports [35]. ACL injuries can lead to knee instability, altered joint kinematics and an elevated risk of long‐term degenerative changes [32]. While structured rehabilitation alone may suffice for selected patients, many opt for surgical reconstruction to restore knee stability and return to sport [17].

ACL reconstruction (ACL‐R) is widely considered the gold standard for restoring mechanical stability in ACL‐deficient knees. However, despite its functional benefits, ACL‐R does not necessarily prevent post‐traumatic osteoarthritis (PTOA) [8]. In fact, several studies have reported that degenerative changes may still occur even after technically successful ACL‐R, prompting ongoing debate regarding the procedure's long‐term protective effects on joint health [5].

Among the compartments affected by PTOA, patellofemoral osteoarthritis (PFOA) has particular clinical relevance. PFOA has been associated with anterior knee pain, reduced function and poorer patient‐reported outcomes (PROs) [45]. Unlike tibiofemoral osteoarthritis (OA), PFOA may be influenced by factors such as the graft choice during ACL‐R [7]. Notably, the incidence of PFOA and risk factors may differ between patients undergoing surgical versus non‐surgical treatment for ACL injuries [27].

This systematic review and meta‐analysis aimed to evaluate whether ACL‐R is associated with a higher likelihood of developing PFOA compared to patients with ACL injuries managed non‐surgically.

## METHODS AND MATERIALS

**Figure 1:**
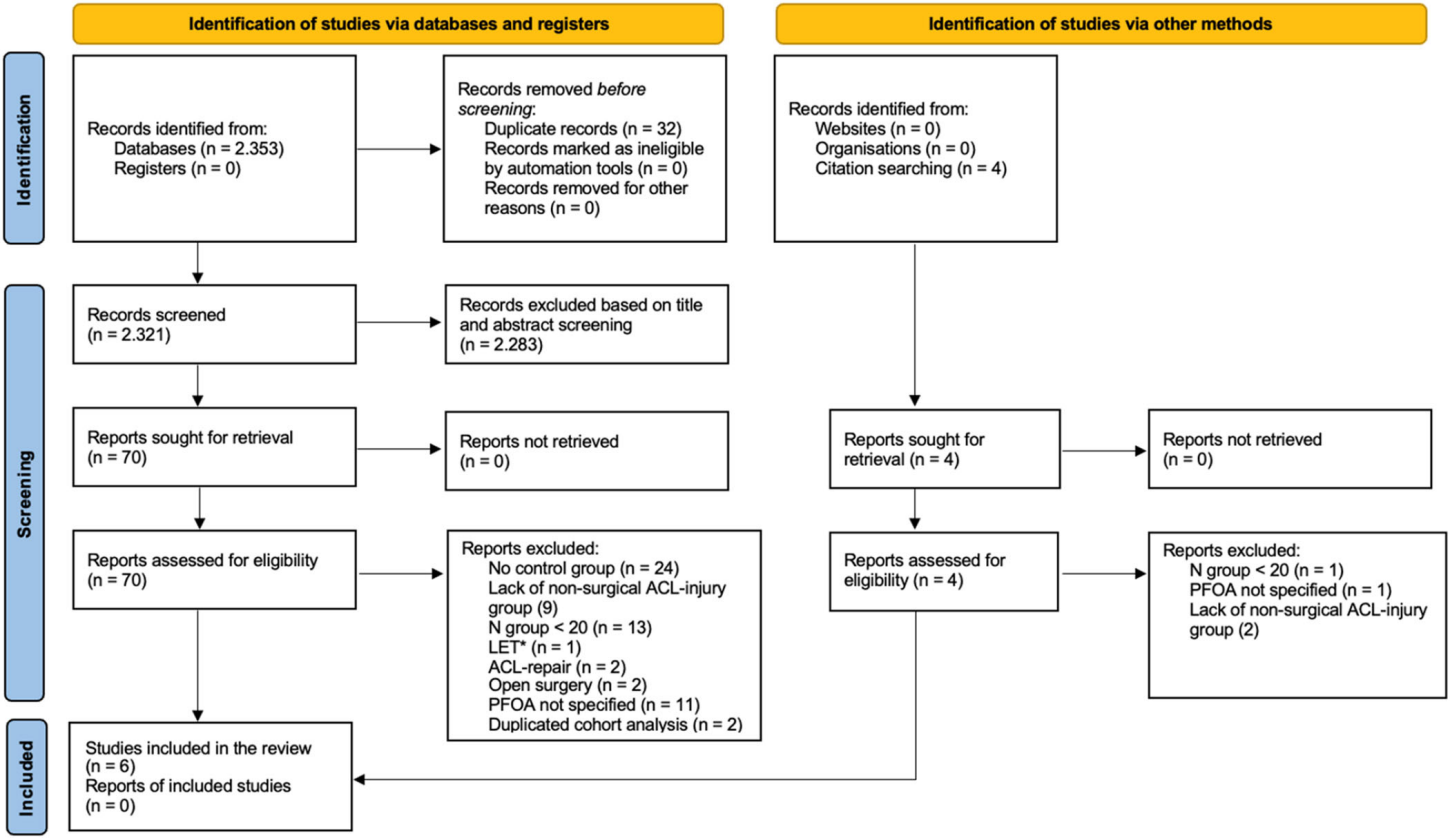
PRISMA (Preferred Reporting Items for Systematic Reviews and Meta‐Analysis) flowchart of the study‐inclusion process. ACL, anterior cruciate ligament; LET, lateral extraarticular tenodesis; *N*, number; PFOA, patellofemoral osteoarthritis.

A comprehensive literature search was conducted across major databases, including PubMed/MEDLINE, Embase, Cochrane Library and Scopus, until June 2025. Details are provided in Supporting Information S1: Table S1. The reporting in this article adhered to the Preferred Reporting Items for Systematic Reviews and Meta‐Analysis (PRISMA) guidelines (Figure 1). The study was registered on PROSPERO: CRD42024571939.

We included only studies in English that involved both patients with ACL injuries undergoing non‐surgical treatment and primary ACL‐R.

Articles published after 1999, reporting exclusively arthroscopic procedures and using either autograft or allograft, with a minimum of 15 patients per study group and a minimum follow‐up of 2 years, were considered. Only studies classified as Level of Evidence 2 and 3, such as randomized controlled trials (RCTs), cohort studies, case‐control studies and cross‐sectional studies, were included.

Studies involving other procedures, such as ACL repair, lateral extra‐articular tenodesis (LET), meniscal transplantations or cartilage restoration procedures, were excluded. Studies that met the above criteria but lacked radiographic or magnetic resonance imaging (MRI) assessment of PFOA were excluded.

All records were imported into a dedicated screening and data extraction platform (Cadima, www.cadima.info). Two independent investigators (D. F. and A. B.) evaluated the studies for the inclusion and exclusion criteria outlined in Table 1. The reference lists of included studies were scrutinized to identify any further relevant sources. In instances of disagreement regarding eligibility criteria, the senior author (C. L.) mediated to resolve discrepancies.

**Table 1:** Inclusion and exclusion criteria.

Inclusion criteria	Exclusion criteria
Patients of any age who underwent arthroscopic primary ACL‐R using either autografts or allografts	Patients who underwent other procedures, such as ACL‐repair, LET, meniscal transplantations or cartilage restoration
Including a control group comprised of non‐surgically ACL injury patients	Studies that failed to report imaging‐based outcomes related to radiographic or MRI evidence of PFOA
Number of patients ≥ 15	<2‐year follow‐up
Level II and III studies	Level IV and V studies
Studies published in English language	

Abbreviations: ACL, anterior cruciate ligament; ACL‐R, anterior cruciate ligament reconstruction; LET, lateral extra‐articular tenodesis; MRI, magnetic resonance imaging; PFOA, patellofemoral osteoarthritis.

Study details (e.g., title, first author, journal, publication year, digital object identifier [DOI], level of evidence and study design), participant characteristics (e.g., age, sex, sample size), specific treatment (e.g., graft types), last follow‐up, PFOA definition and number of patients with/without PFOA, clinical outcomes and additional injuries were extracted. In cases where PFOA was assessed at various time points, estimates from the last available follow‐up were considered.

The quality of the studies was assessed using a modified version of the Coleman Methodology Score (MCMS) [6]. For randomized trials, the risk of bias was assessed using Version 2 of the Cochrane risk‐of‐bias tool (RoB 2), while for non‐randomized cohort and case‐control studies the risk of bias was analysed by the Risk of Bias in Non‐randomized Studies of Interventions (ROBINS‐I). The potential biases were categorized as low, moderate or some concerns and high, based on established questions.

The following PFOA thresholds used in the articles were considered: Kellgren and Lawrence (KL) Grade ≥ 2 (one study), Osteoarthritis Research Society International Osteoarthritis Research Society International (OARSI) Grade ≥ 2 (two studies) and International Knee Documentation Committee (IKDC) Grade > B (two studies). One study used an unconventional grade system for PFOA definition, divided into four grades and the X‐ray threshold for OA grade mild or more was also considered in terms of severity.

Categorical data were analysed using the *χ* ^2^ test or Fisher's exact test. A meta‐analysis based on a random‐effects model was performed using R 4.4.2 (R Foundation for Statistical Computing) to calculate the pooled odds ratio (OR) of PFOA in patients following ACL‐R compared to patients with ACL injury and non‐surgical treatment. Heterogeneity tests were interpreted as follows: *I* ^2^ < 40%, low heterogeneity; 30% > *I* ^2^ < 60%, moderate heterogeneity; 50% > *I* ^2^ < 90% substantial and *I* ^2^ > 75%–100%, considerable heterogeneity [20].

## RESULTS

A total of 2353 records were identified through the initial database search. After removing 32 duplicate records, 2321 records were screened. Of these, 2283 were excluded based on title and abstract screening, and 70 articles then underwent the full‐text screening. After the assessment, 63 articles were excluded for various reasons: 24 studies lacked of any control group, 13 had a sample size below 10, 11 studies did not specifically assessed PFOA, 9 studies were without nonoperative ACL injury group, 1 study involved LET, 1 study focused on ACL repair, 2 articles involved ACL‐R through an open procedure and 3 articles had the same cohort and was considered only the most recent article (Figure 1).

Ultimately, six studies met the inclusion criteria and were included in the systematic review (Figure 1). These six studies comprised 589 participants (308 male, 281 female; mean age = 28 ± 3.24 years; mean follow‐up period = 11.33 years [6.6–15.7 years]), including 306 patients with ACL‐R and 283 with ACL injury and non‐surgical treatment. The included studies consisted of one RCT [41], one non‐RCT [16], one secondary analysis of a prospective trial [15] and three cohort studies [26, 33, 38], published between 2004 and 2023.

Regarding the choice of graft, an autologous bone–patellar tendon–bone (BPTB) graft was used in two studies [16, 38]. One study implied hamstring (HT) [41], while different combinations of BPTB and HT were used in two studies [15, 26]. In one article, the graft choice was not clearly specified; it mentioned the use of BPTB graft in 65% of cases, while in the remaining 35%, it was not disclosed [33].

In one article, the control group included both nonoperative ACL injury patients and their contralateral knees; however, only the first group was used for analysis [33]. PROs were presented in all the included articles; three studies reported Knee Injury and Osteoarthritis Outcome Score (KOOS). The Lysholm score was reported in two studies, whereas the Tegner score was considered in four articles. A 36‐Item Short Form Survey (SF‐36) was evaluated in two studies, and IKDC was assessed in one study. One article presented information about the Quadriceps and Hamstring Strength Index. Regarding concomitant injuries, meniscal tears were recorded in two articles and meniscal procedures, such as meniscectomy or meniscal repair, were reported in four articles. Two studies did not report any details regarding meniscal injury or related procedures. The characteristics of the included studies are summarized in Tables 2 and 3.

**Table 2:** Demographic characteristics of the studies included in the systematic review.

Study	Study design	Level of evidence	Sex (M/F)	Age (mean ± SD)	BMI (kg/m^2^) ± SD	*N* (study)	Type of control group	*N* (control)	N (intervention)	Graft type	Follow‐up time (years)
Neuman et al. [38]	Cohort study	III	31/44	(15–43)	N/A	75	ACL‐tear + rehab	60	15	BPTB (autograft)	15.7
Fithian et al. [16]	Prospective non‐randomized controlled clinical trial	II	101/108	(16–69)	N/A	209	ACL‐tear + rehab	113	96	BPTB (autograft)	6.6
Lohmander et al. [33]	Cohort study	III	0/67	31 ± 6	23	67	‘ACL‐tear + rehab; contralateral knee’	26	41	BPTB 65%, no mentioned 35%	12
Keays et al. [26]	Cohort study	III	69/32	25 ± 7.25 (23–49)	N/A	101	ACL‐tear + rehab	45	56	BPTB and HT	11
Tsoukas et al. [41]	Randomised controlled trial	II	32/0	32 (20–39)	N/A	32	ACL‐tear + rehab	15	17	HT	10.1
Filbay et al. [15]	Secondary analysis of a prospective trial	III	75/30	26 ± 5	25.2	105	ACL‐tear + rehab	24	81	BPTB and HT	11

Abbreviations: ACL, anterior cruciate ligament; BMI, body mass index; BPTB, bone–patellar tendon–bone autograft; Control, ACL injury + Rehab, anterior cruciate ligament injury group with non‐surgical treatment; HT, hamstring autograft; Intervention, anterior cruciate ligament reconstruction; N/A, not applicable.

**Table 3:** Outcome characteristics of the included studies.

Study	Study design	Level of evidence	Sex (M/F)	Age (mean ± SD)	BMI (kg/m^2^) ± SD	*N* (study)	Type of control group	*N* (control)	*N* (intervention)	Graft Type	Follow‐up time (years)
Neuman et al. [38]	Cohort study	III	31/44	26 (15–43)	N/A	75	ACL‐tear + rehab	60	15	BPTB (autograft)	15.7
Fithian et al. [16]	Prospective non‐randomized controlled clinical trial	II	101/108	(16–69)	N/A	209	ACL‐tear + rehab	113	96	BPTB (autograft)	6.6
Lohmander et al. [33]	Cohort study	III	0/67	31 ± 6	23	67	‘ACL‐tear + rehab; contralateral knee’	26	41	BPTB 65%, no mentioned 35%	12
Keays et al. [26]	Cohort study	III	69/32	25 ± 7.25 (23–49)	N/A	101	ACL‐tear + rehab	45	56	BPTB and HT	11
Tsoukas et al. [41]	Randomised controlled trial	II	32/0	32 (20–39)	N/A	32	ACL‐tear + rehab	15	17	HT	10.1
Filbay et al. [15]	Secondary analysis of a prospective trial	III	75/30	26 ± 5	25.2	105	ACL‐tear + rehab	24	81	BPTB and HT	11

Abbreviations: ACL, anterior cruciate ligament; ACL injury + Rehab, anterior cruciate ligament injury group with non‐surgical treatment; BMI, body mass index; BPTB, bone–patellar tendon–bone autograft; HT, hamstring autograft; ICOAP, Intermittent and Constant Osteoarthritis Pain Score; IKDC, The International Knee Documentation Committee score; JSN, Joint Space Narrowing; KOOS, The Knee Injury and Osteoarthritis; Outcome Score; KL, Kellgren–Lawrence classification; N/A, not applicable; OA, osteoarthritis; OARSI atlas, Osteoarthritis Research Society International classification; PFOA, patellofemoral osteoarthritis; SF‐36, 36‐Item Short Form Survey.

**Figure 2:**
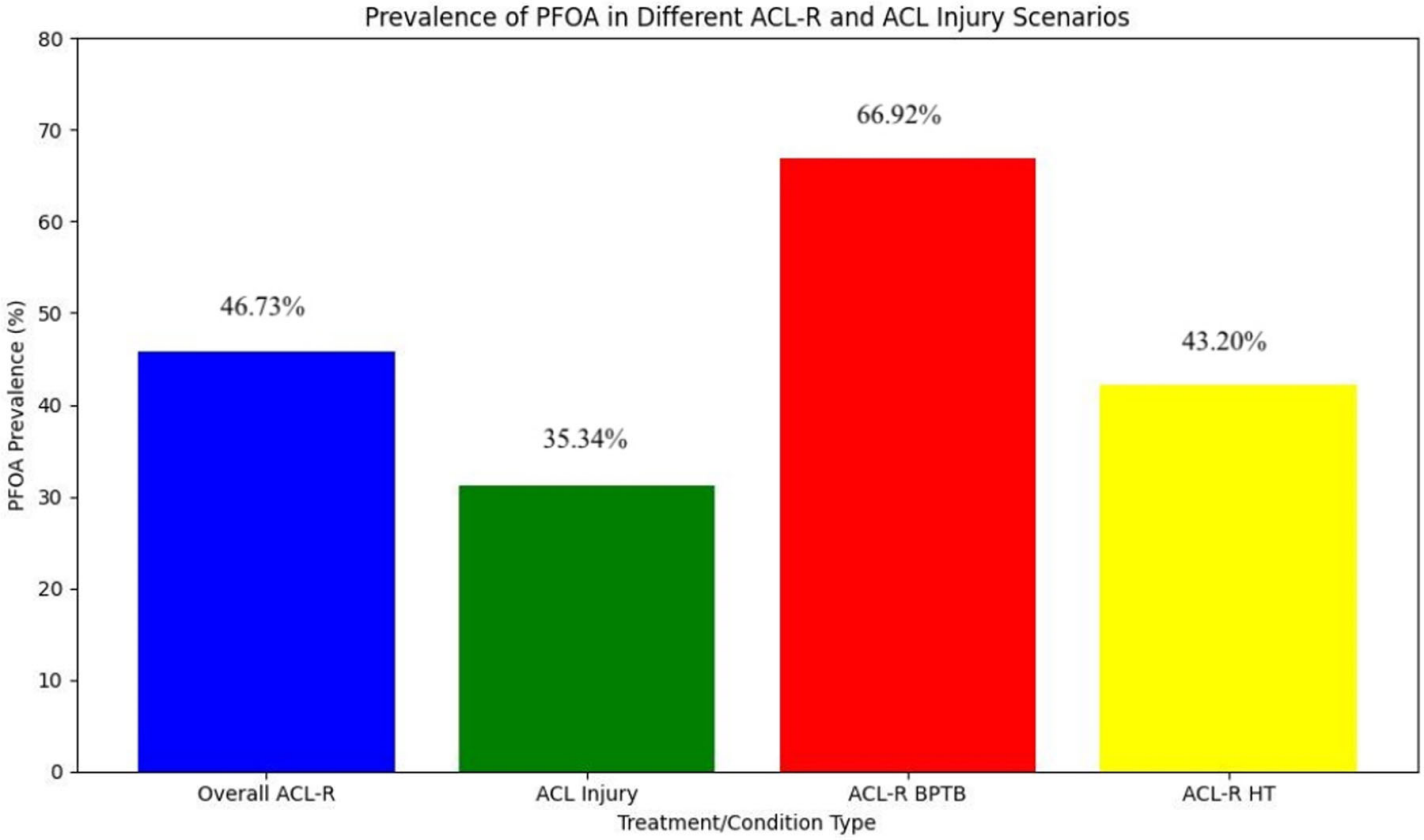
Patellofemoral osteoarthritis prevalence. ACL injury, anterior cruciate ligament injury and non‐operative treatment group; ACL‐R, anterior cruciate ligament reconstruction; BPTB, bone–patellar tendon–bone autograft; HT, hamstring autograft; PFOA, patellofemoral osteoarthritis.

The pooled prevalence of PFOA in patients who underwent ACL‐R was 46.73% (35.71%−70.83%), and 35.34% (3.85%–60.00%) in patients undergoing non‐surgical treatment. In patients undergoing ACL‐R with a BPTB autograft (11.2 years follow‐up), the pooled prevalence of PFOA was 66.92% (95% confidence interval [CI]: 46.67%–70.83%). For patients with a HT autograft (10.6 years follow‐up), the prevalence was 43.20% (29.63%–64.70%). The BPTB group showed a significantly higher prevalence of PFOA compared to the HT group (*p* < 0.01) (Figure 2).

Male patients demonstrated a higher association with PFOA than females in both the ACL‐R group (34.38% vs. 7.77%, *p* < 0.001) and the non‐surgical ACL injury group (28.13% vs. 0.97%, *p* < 0.001).

### Risk of bias

**Figure 3:**
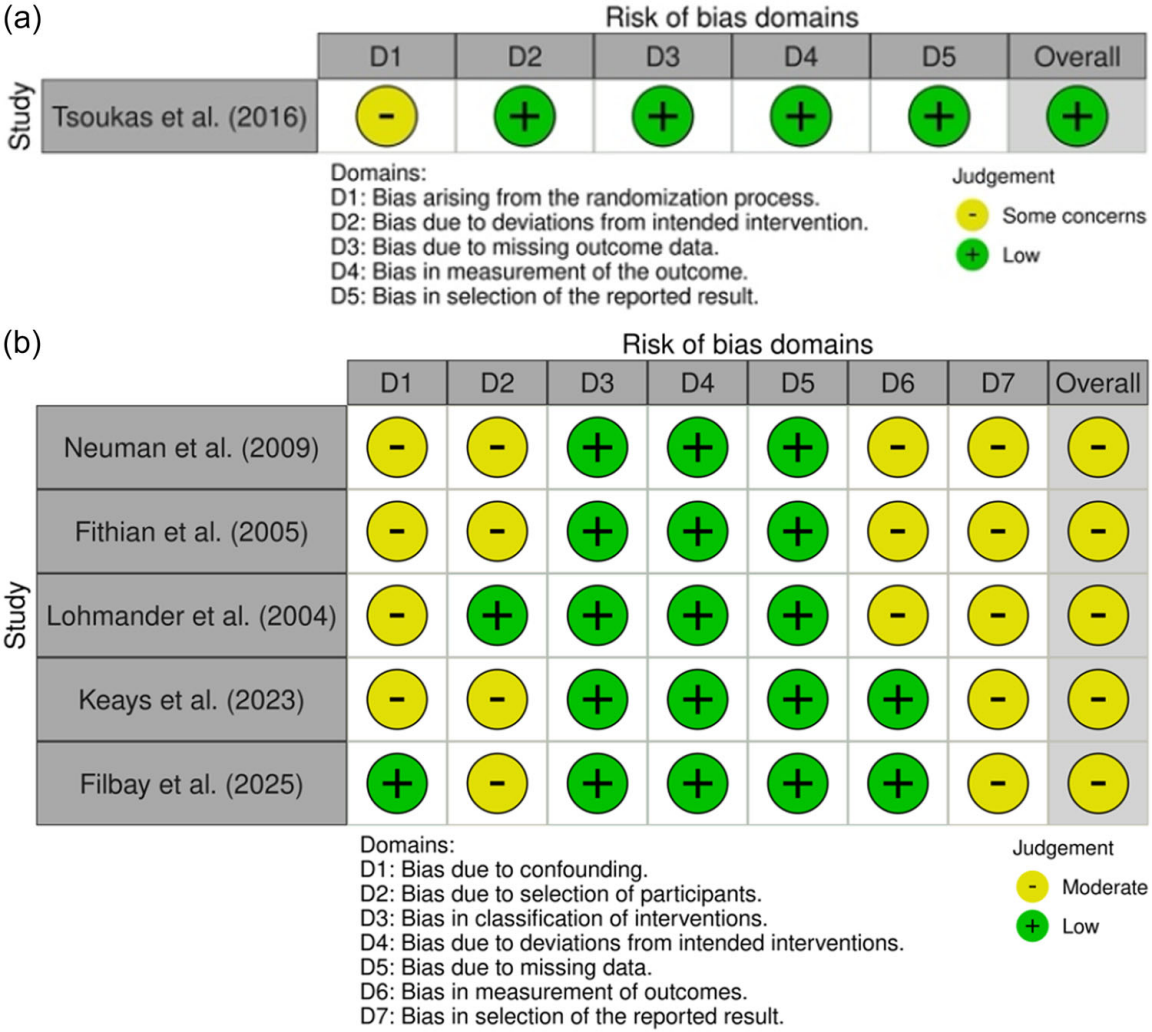
Risk of bias for included randomized controlled trials (a) and non‐randomized controlled trials (b).

The only included RCT had a low risk of bias, with some concern arising from the randomization process. Non‐randomized studies demonstrated an overall moderate risk of bias, particularly in areas related to confounding, participant selection, outcome measurement and reported results. The risk of bias assessment is summarized in Figure 3.

### Quality assessment

The MCMS ranged from 73 to 85, reflecting a moderate quality level. The most common limitation identified was the lack of clarity in the description of rehabilitation protocols, as well as the variability in the number and description of the surgical procedures (Table 4).

**Table 4:** Modified Coleman Methodology Score (MCMS).

References	Part A							Part B			Part A	Part B	Total MCMS
	Study size	Follow‐up	Number of procedures	Study type	Diagnostic certainty	Description of procedure	Description of rehabilitation	Outcome criteria	Outcome assessment	Selection process			
Neuman et al. [38]	10	5	5	5	5	5	5	10	8	15	40	33	73
Fithian et al. [16]	10	5	5	10	5	10	5	8	12	15	50	35	85
Lohmander et al. [33]	10	5	10	5	5	5	5	8	11	15	45	34	79
Keays et al. [26]	10	5	10	5	5	10	5	8	7	15	50	30	80
Tsoukas et al. [41]	4	5	5	15	5	10	5	8	7	15	49	30	79
Filbay et al. [15]	10	5	10	5	5	10	5	8	7	15	50	30	80

### Meta‐analysis

**Figure 4:**
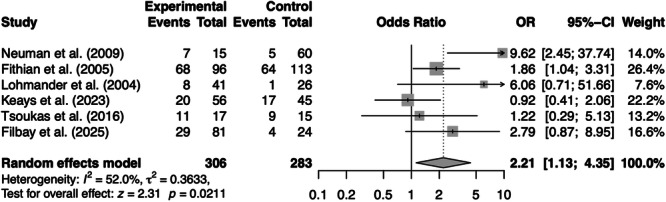
Forest plot for the prevalence of patellofemoral osteoarthritis following anterior cruciate ligament (ACL) reconstruction compared to ACL injury and non‐operative treatment group. CI, confidence interval; OR, odds ratio; *p* = *p* value; *τ* ^2^ = variance of the true effects.

**Figure 5:**
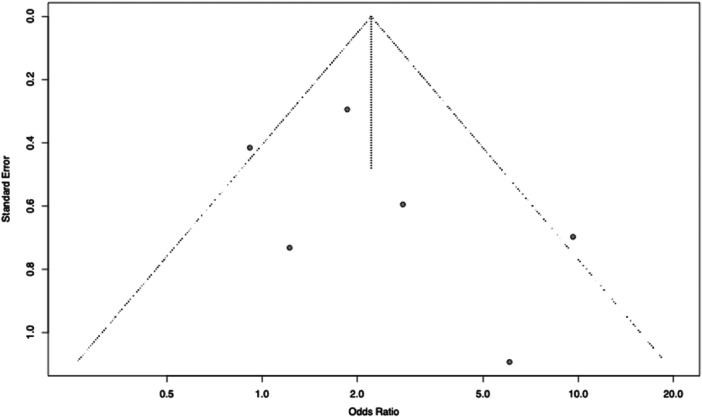
Funnel plot of odds ratios for the prevalence of patellofemoral osteoarthritis among included studies following anterior cruciate ligament (ACL) reconstruction compared to the ACL injury and non‐operative treatment group.

The pooled OR for developing PFOA was statistically significant in patients who underwent ACL‐R compared to a non‐surgical ACL injury group, OR = 2.21 (95% CI: 1.13–4.35, *p* = 0.02). There was moderate heterogeneity across the included studies (*I* ^2^ = 52.0%) (Figures 4 and 5).

## DISCUSSION

This systematic review and meta‐analysis demonstrated that patients undergoing ACL‐R are significantly more likely to develop PFOA compared to those non‐surgically managed after an ACL injury (OR = 2.21). Within the ACL‐R cohort, patients reconstructed with BPTB autograft exhibited a significantly higher prevalence of PFOA than those with HT autograft (*p* < 0.01). Moreover, male patients showed a significantly higher association with PFOA in both ACL‐R and non‐surgical ACL injury groups. These findings indicate that individuals who underwent ACL‐R had more than twice the odds of developing PFOA compared to patients who received only rehabilitation after an ACL injury. Moreover, the risk of PFOA is significantly greater in males and in surgical patients, particularly with BPTB autograft.

Our article aligns with the current literature, confirming that ACL‐R, despite restoring knee stability, is associated with the development of PFOA [2, 4]. This may be due to several factors that contribute to PFOA following ACL injury and ACL‐R.

Altered patellofemoral joint (PFJ) kinematics, which may result from graft tunnel malpositioning during ACL‐R, can modify the contact area on the patellofemoral cartilage, thereby promoting cartilage degeneration [9, 31, 42]. Additionally, inadequate or delayed postoperative rehabilitation can lead to abnormal loading of the PFJ and increase the risk of PFOA following ACL‐R [19]. Quadriceps hypotrophy still is a widespread phenomenon following surgery, particularly when rehabilitation is suboptimal or if anterior knee pain limits quadriceps engagement due to donor site morbidity, as seen more often in BPTB autografts [10, 11, 14, 36, 40].

The selection of graft appears to be an unclear risk factor for developing PFOA. We included articles that specified the graft used, of which only one [26] examined cartilage degeneration based on the different autografts employed. This review revealed a significantly higher association of PFOA in BPTB autograft compared to HT following ACL‐R. The findings are consistent with previous research, which indicates a stronger association between PFOA and ACL‐R when using BPTB autograft, potentially due to altered joint biomechanics and patellar tracking [16, 25, 30, 39]. In contrast, Barenius et al. showed that the HT autograft is a major contributor to PFOA compared to BPTB [2]. Harvesting HT may disrupt the balance of posterior chain muscles, leading to compensatory mechanisms that could compromise dynamic knee stability and alter PFJ, thereby increasing cartilage degeneration [37]. Lastly, a recent publication by Lucidi et al. found no significant differences in PFJ degeneration between graft types at 20‐year follow‐up [34].

Sex‐based differences in PFOA, particularly in the context of ACL injury and ACL‐R, underlying factors may contribute to this prevalence disparity. Males typically exhibit greater quadriceps dominance, which may lead to increased PFJ loading and cartilage degeneration over time [13]. In contrast, females may rely more on hip and hamstring musculature, potentially distributing joint loads differently and reducing PFJ degeneration following ACL‐R [7]. The knee inflammatory status following ACL injury and surgery could also differ between sexes, influencing PFOA development. The acute inflammation response following ACL‐R leads to the release of cytokines (e.g., interleukin‐1 beta [IL‐1β], interleukin‐6 [IL‐6], tumor necrosis factor‐alpha [TNF‐α]) and matrix metalloproteinases into the synovial fluid, which degrade the cartilage matrix and harm chondrocytes [23, 24]. Emerging evidence suggests that sex hormones, such as estrogen, downregulate inflammatory pathways in female animals, potentially offering a protective effect against cartilage catabolism [12].

Other biological factors may also influence the progression of PFOA, regardless of sex differences. Persistent knee synovitis and low‐grade inflammation in both ACL‐R and non‐surgical ACL‐injury patients perpetuate cartilage catabolism and contribute to PFOA development [3, 22, 24, 28]. Additionally, in ACL‐R, the surgical procedure may exacerbate this inflammatory response due to tissue disruption (second hit), thereby accelerating cartilage loss in susceptible individuals [1, 43]. Instead, in the non‐surgical ACL injury group, PFOA prevalence may continue to be driven by the chronic low‐level inflammation status resulting from the initial injury, and recurrent microtrauma during activity due to instability, which may accelerate cartilage degeneration [18, 21, 29].

Interpreting the likelihood of PFOA between ACL‐R and non‐surgical groups is complicated by several limitations. First, in this study, PFOA refers to image‐based findings and does not necessarily reflect symptomatic OA. Our stringent inclusion criteria enhanced the study's quality but resulted in a reduced number of eligible studies, which may limit the generalizability of our findings. Additionally, the pooled nature of the data implies heterogeneity across different population ages and follow‐up times. Data from early time points when structural degenerative changes in the knee are less prevalent may underestimate the odds of PFOA. Third, this review assessed PFOA using different threshold values derived from the X‐ray technique. Comparing results across studies is challenging due to the different OA classification systems employed. For instance, Filbay et al. [44] and Neuman et al. [38] utilized the OARSI classification to evaluate PFOA, highlighting a notable discrepancy, as this method is more sensitive in detecting OA than radiographic assessments that rely on KL classifications. Given the limited number of included studies, incorporating those using a uniform OA classification system was not feasible. Ultimately, the treatment following an ACL injury was influenced by both the patient's preferences and the recommendations from the surgical team. In two studies [15, 16], patients who were unresponsive to non‐surgical methods proceeded with ACL‐R, thus joining the surgery group. Conducting a study with non‐surgical patients who experience knee instability and need surgery is challenging due to ethical considerations and recruitment issues, as reported by Filbay et al. [15]. Our data suggest that patients with a stable knee following ACL‐R are more likely to develop PFOA. This could be due to resuming high‐impact sports, potentially increasing mechanical demands on the patellofemoral compartment. In contrast, those managed non‐operatively may be limited in their sports activities. However, while the observed PFOA may reflect altered activity profiles, our findings cannot definitively confirm or refute this claim, as detailed activity tracking data is unavailable. Moreover, most of the included papers did not provide data on specific physiotherapy protocols or the timing and duration of rehabilitation measures. Similarly, the interval between injury occurrence and surgical intervention was rarely reported, despite its known relevance to joint outcomes. Finally, the ACL‐R technique used and the accuracy of anatomical femoral footprint restoration were also insufficiently documented. The lack of these data restricts the ability to control for confounding factors, and consequently, the conclusions must be viewed with caution, as unreported variability in these parameters might have influenced the observed outcomes.

## CONCLUSIONS

This meta‐analysis demonstrated that ACL‐R significantly increases the likelihood of PFOA compared to non‐surgical ACL treatment, especially when using BPTB autografts and in male patients. These findings highlight the potential detrimental effects of ACL‐R on PFJ degeneration, emphasizing the necessity for targeted interventions to mitigate PFOA. Targeted preventive strategies, including optimized graft selection and enhanced rehabilitation protocols, are crucial for reducing the risk of PFOA following ACL‐R. However, these results should be interpreted with caution, and further studies are needed to clarify the contribution of ACL injury and ACL‐R to the development of PFOA.

## AUTHOR CONTRIBUTIONS

*Conceptualization*: Domenico Franco. *Methodology*: Domenico Franco. *Formal analysis and investigation*: Domenico Franco and Alexander Bumberger. *Writing draft preparation*: Domenico Franco. *Review*: Alexander Bumberger, Chilan B. G. Leite, Sebastian Schmidt, Fabrizio Russo, Gianluca Vadalà, Rocco Papalia, Vincenzo Denaro, Cale Jacobs and Christian Lattermann. *Editing*: Domenico Franco. *Supervision*: Rocco Papalia, Vincenzo Denaro, Cale Jacobs and Christian Lattermann.

## CONFLICT OF INTEREST STATEMENT

The authors declare no conflict of interest.

## ETHICS STATEMENT

The authors have nothing to report.

## Supporting information

SUPPLEMENTAL MATERIAL.

## Data Availability

Ready to share.
