# Supplementary material for: Comparison of patellofemoral osteoarthritis between patients treated surgically or non‐surgically following anterior cruciate ligament injuries: A systematic review and meta‐analysis
Source: J Exp Orthop. 2026 May 18;13(2):e70697. doi: 10.1002/jeo2.70697 (PMC13181591; doi:10.1002/jeo2.70697)
Supplement: Supplementary file 1 — SUPPLEMENTAL MATERIAL. [file JEO2-13-e70697-s001.docx]

**SUPPLEMENTAL MATERIAL**

**Supplementary Table 1. Search terms.**

**
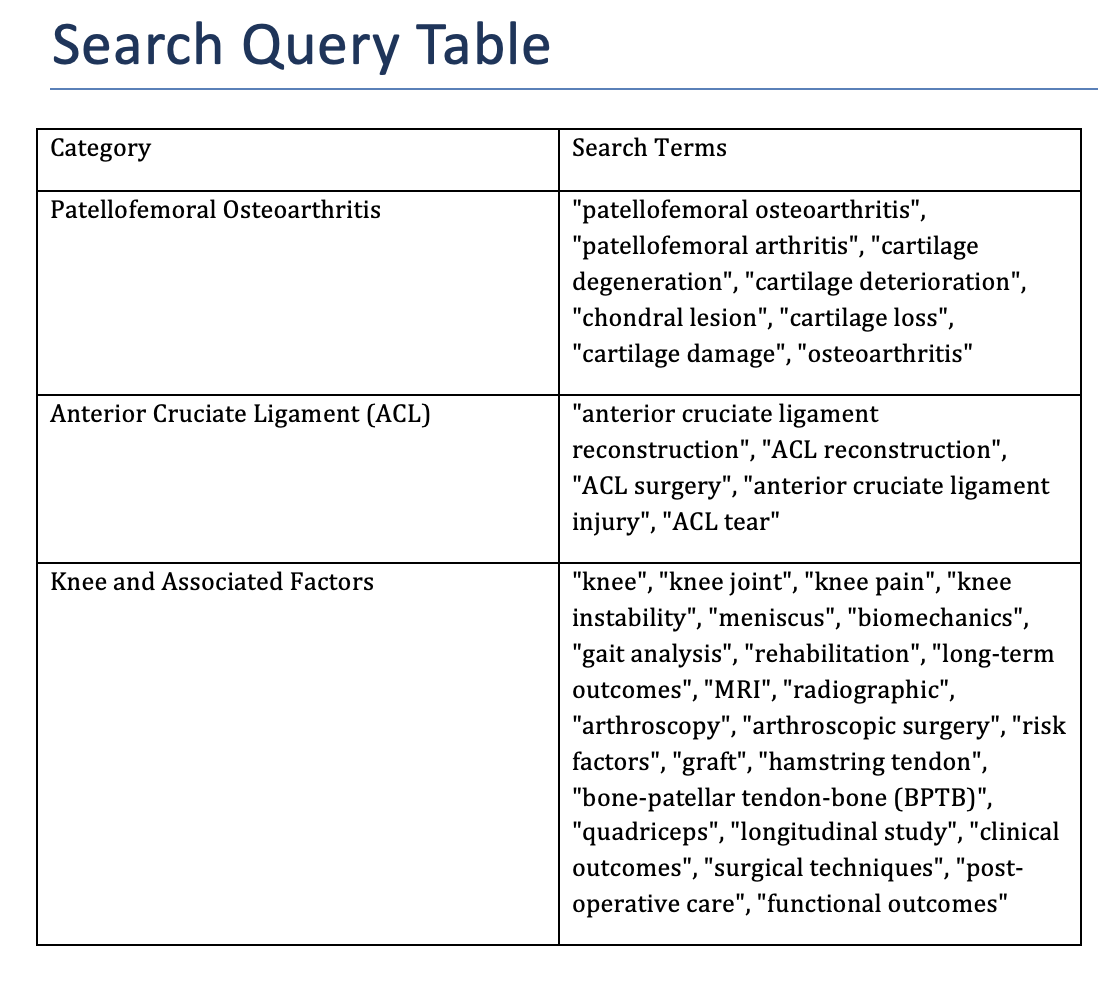
**

(Patellofemoral osteoarthritis OR patellofemoral arthritis OR cartilage degeneration OR chondral lesion OR cartilage loss OR cartilage damage OR osteoarthritis) AND (anterior cruciate ligament reconstruction OR ACL reconstruction OR ACL surgery OR anterior cruciate ligament injury OR ACL tear) AND (knee OR knee joint OR knee pain OR knee instability OR meniscus OR biomechanics OR gait analysis OR rehabilitation OR long-term outcomes OR MRI OR radiographic OR arthroscopy OR arthroscopic surgery OR risk factors OR graft OR hamstring tendon OR bone-patellar tendon-bone OR quadriceps OR longitudinal study OR clinical outcomes OR surgical techniques OR post-operative care OR functional outcomes)
